# High risk HPV-positive women cervicovaginal microbial profiles in a Greek cohort: a retrospective analysis of the GRECOSELF study

**DOI:** 10.3389/fmicb.2023.1292230

**Published:** 2023-11-30

**Authors:** Electra Sofou, Glykeria Gkoliou, Nikolaos Pechlivanis, Konstantinos Pasentsis, Kimon Chatzistamatiou, Fotis Psomopoulos, Theodoros Agorastos, Kostas Stamatopoulos

**Affiliations:** ^1^Institute of Applied Biosciences, Centre for Research and Technology Hellas, Thessaloniki, Greece; ^2^Department of Molecular Biology and Genetics, Democritus University of Thrace, Alexandroupoli, Greece; ^3^Department of Genetics, Development and Molecular Biology, Faculty of Biology, Aristotle University of Thessaloniki, Thessaloniki, Greece; ^4^1st Department of Obstetrics and Gynecology, Aristotle University of Thessaloniki, Papageorgiou General Hospital, Thessaloniki, Greece; ^5^St. Luke’s Hospital, Thessaloniki, Greece; ^6^Department of Molecular Medicine and Surgery, Karolinska Institute, Stockholm, Sweden

**Keywords:** HPV, vaginal microbiome, cervical microbiome, next generation sequencing (NGS), *Lacticaseibacillus*

## Abstract

Increasing evidence supports a role for the vaginal microbiome (VM) in the severity of HPV infection and its potential link to cervical intraepithelial neoplasia. However, a lot remains unclear regarding the precise role of certain bacteria in the context of HPV positivity and persistence of infection. Here, using next generation sequencing (NGS), we comprehensively profiled the VM in a series of 877 women who tested positive for at least one high risk HPV (hrHPV) type with the COBAS^®^ 4,800 assay, after self-collection of a cervico-vaginal sample. Starting from gDNA, we PCR amplified the V3–V4 region of the bacterial 16S rRNA gene and applied a paired-end NGS protocol (Illumina). We report significant differences in the abundance of certain bacteria compared among different HPV-types, more particularly concerning species assigned to *Lacticaseibacillus, Megasphaera* and *Sneathia* genera. Especially for *Lacticaseibacillus*, we observed significant depletion in the case of HPV16, HPV18 versus hrHPVother. Overall, our results suggest that the presence or absence of specific cervicovaginal microbial genera may be linked to the observed severity in hrHPV infection, particularly in the case of HPV16, 18 types.

## Introduction

Infection with the human papillomavirus (HPV) is one of the most common sexually transmitted infections (STIs) worldwide (Schiffman et al., 2016). Although the vast majority of infections resolve without disease progression, a smaller -yet significant- number of infected individuals develop HPV-associated cancer. In fact, HPV accounts for about 600,000 new cancer cases annually and is considered as the main cause of invasive cervical cancer (ICC); moreover, it is implicated in many other cancers, including oropharyngeal cancers (OPCs) and anal cancer (Chaturvedi et al., 2011; Forman et al., 2012; Roden and Stern, 2018). Several genotypes of HPV have been associated with high-risk infections, i.e., 16, 18, 31, 33, 35, 39, 45, 51, 52, 56, 58, and 59 (Reid et al., 1982; Muñoz et al., 2003). HPV16 and 18 are the most common carcinogenic (high-risk) HPV types involved in cervical cancer pathogenesis (Reid et al., 1982; Muñoz et al., 2004), responsible for low-grade squamous intraepithelial lesions (LSIL), high-grade squamous intraepithelial lesions (HSIL), adeno- and squamous cell carcinoma (SCC) (Lazare et al., 2019). Collectively, HPV16 and HPV18 infections account for ~70% of cervical cancer cases worldwide (Pimple and Mishra, 2022).

An increasing number of studies support the significant role of the vaginal and the cervical microbiome in HPV infection persistence and the formation of precancerous lesions in the cervix (Cervical Intraeptithelial Neoplasia, CIN) (Mirabello et al., 2016; Cheng et al., 2020; Lin et al., 2022). The healthy, cervicovaginal microbial flora is normally dominated by *Lactobacillus* spp., a genus known for its key role in the maintenance of the cervicovaginal eubiosis by producing bacteriocins, lactic acid and hydrogen peroxide, exerting a protective effect on the cervix/vagina from pathogen infections (Ravel et al., 2011; Lebeau et al., 2022). However, HPV infections can disturb the balance of vaginal and cervical microenvironment, leading to abnormal changes in microbiota, including *Lactobacillus* spp. depletion and predominance of *Lactobacillus iners* and anaerobic bacteria (such as *Gardnerella, Prevotella, Sneathia, Megasphaera*) (Lee et al., 2013; Bik et al., 2019; Borgogna et al., 2020; Caselli et al., 2020; Usyk et al., 2020; Wei et al., 2021; Lebeau et al., 2022). These bacteria may destroy the cervical epithelial barrier and promote HPV infection by producing enzymes and metabolites which act on several cellular pathways that enable persistence of viral infection and disease progression (Li et al., 2012).

Studies have demonstrated that abnormal changes in the composition of the vaginal microbiome, which lead to reduced mucus production and consequent clearance of pathogenic bacteria, can be associated with the late clearance of HPV infection and progression to CIN (Watts et al., 2005; Brotman et al., 2014; Audirac-Chalifour et al., 2016; Lin et al., 2022). Furthermore, the development of squamous intraepithelial lesions (SIL) has been associated with an increased vaginal microbial diversity (Curty et al., 2020; Lin et al., 2022). Altogether, all these findings allude to a significant role of the cervicovaginal microbiome in HPV-associated CIN. However, to-date there are no direct links of specific genera/species to the infection by specific HPV types or to the development of CIN.

Here, we investigated the vaginal and cervical microbiota composition and its potential role in HPV infection, employing amplicon-based Next Generation Sequencing (NGS) of cervicovaginal specimens from a large series of HPV-infected women. Our aim was to characterize in detail the resident cervical and vaginal microbial flora under conditions of HPV infection and their potential link to CIN.

## Materials and methods

### Study cohort and design of the study

This is a retrospective study analyzing samples from a cohort of 877 non-pregnant women who were diagnosed with high-risk (hr) HPV infection in the context of GRECOSELF, a nationwide observational cross-sectional study aiming to suggest a way to implement HPV-DNA testing in conjunction with self-sampling for cervical cancer screening in Greece (Agorastos et al., 2019).

In brief, upon written informed consent, cervico-vaginal samples were self-collected with a vaginal dry swab (Roche Molecular Systems) and then stored and transported as previously described (Agorastos et al., 2019). HPV infection was confirmed by the detection of viral DNA using the Cobas 4,800 HPV test (Roche, United States). This assay is specifically designed to identify 14 hrHPVs, namely HPV16, 18, 31, 33, 35, 39, 45, 51, 52, 56, 58, 59, 66, 68 and also performs partial genotyping of the HPV16, HPV18 types among the other hrHPV types.

Women who tested positive for at least one hrHPV type were referred for colposcopy. Overall, we acquired colposcopic data from 636/877 (72.5%) women, whereas the remainder 241/877 (27.5%) for various reasons did not comply with their colposcopy referral upon their HPV test positive result. In cases with abnormal or suspicious colposcopic findings, multiple focal biopsies, and/or endocervical curettage (ECC), were taken from the abnormal area of the cervix. ECC was performed in case of a transformation zone type III.

Histopathological examination was performed by expert histopathologists. Based on the grade of CIN, cases were classified in groups, namely (from low grade to high grade CIN) CIN1 < CIN2 < CIN3 or worse (CIN3+). Women with no evidence of CIN, were classified as ‘No detectable disease (NDD)’ in terms of cervical histopathologic findings.

**Figure 1:**
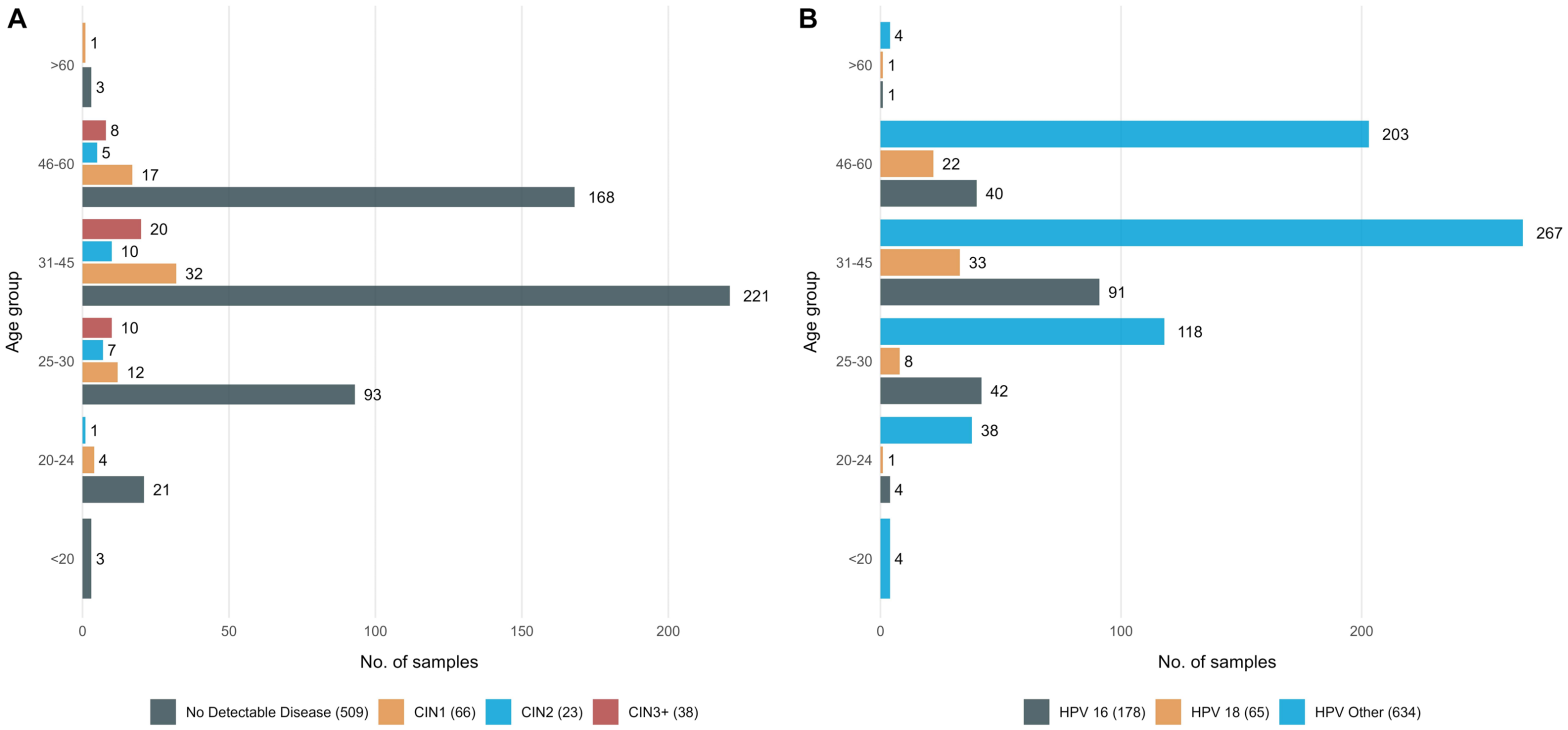
Detailed information on the study group **(A)** 636 women classified in 4 groups based on their histopathologic findings and age group, **(B)** all 877 women who tested positive with the Cobas assay classified in 4 groups, based on the HPV type responsible for the infection and age group.

Samples analyzed for this study included 877 self-collected vaginal samples, each corresponding to a different woman, who tested positive for either HPV16, or HPV18 types or another high-risk type, namely “HPV other” (Figure 1). Moreover, for 60/877 women we analyzed paired samples, one self-collected (vaginal) and one collected by a physician prior to colposcopy (cervico-vaginal).

### Ethical approval and clinical data management

All procedures were in accordance with the standards set by the 1964 Declaration of Helsinki, and were also approved by the Ethics Committees of the Aristotle University of Thessaloniki and the Centre for Research and Technology Hellas. Next Generation Sequencing, as well as clinical data were stored and handled in accordance with the ISO/IEC 27001:2013 guidelines.

### 16S V3–V4 amplification and high-throughput sequencing methodology

Starting with 12.5 ng of total DNA as template, we PCR amplified the V3–V4 region of the bacterial 16S rRNA gene using primers with overhang adapters (forward primer with overhang adapter).

5’-TCGTCGGCAGCGTCAGATGTGTATAAGAGACAGCCTACGGGNGGCWGCAG-3′; reverse primer with overhang adapter.

5’-GTCTCGTGGGCTCGGAGATGTGTATAAGAGACAGGACTACHVGGGTATCTAATCC-3′.

Both the amplicon PCR, as well as the index PCR reactions were carried out using the KAPA HiFi HotStart DNA polymerase 2X ReadyΜix (Roche, United States) and SYTO9 (Thermo Fisher Scientific, United States) to monitor the amplification in real-time. Regarding thermal cycling conditions, 16S amplification was initiated by denaturation at 98°C for 20s, followed by 30 cycles of denaturation at 98°C for 20s to annealing of the primers at 55°C for 30s, and 72°C for 30s with a final extension at 72°C for 5 min.

NGS libraries were prepared by direct incorporation of unique index primer pairs (Nextera XT, Illumina) to 1 μL of the adapter tailed PCR amplicons. Thermal cycling conditions included denaturation at 98°C for 2 min, followed by 10 cycles of ramping from 98°C for 15 s to 64°C for 60 s. Paired-end NGS was performed with the MiSeq reagent v3 kit (2 × 300 bp) in a MiSeq Benchtop Sequencer (Illumina, United States) according to the manufacturer’s instructions.

### Bioinformatics, statistical analysis, and data visualization

**Figure 2:**
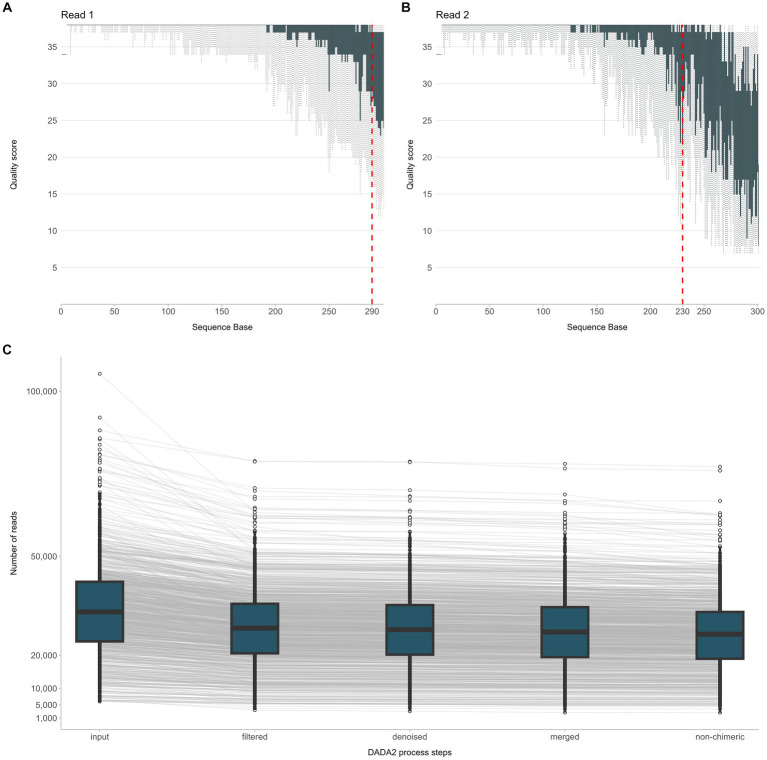
Overview of the NGS raw data processing steps from quality filtering of sequencing reads R1, R2 **(A,B)** to quality assessment of the joined reads and exclusion of chimeric sequences **(C)**.

NGS raw data were analyzed using the QIIME 2020.8.0 software (Bolyen et al., 2019). In detail, adapter trimming of the demultiplexed paired-end sequences was performed using the Cutadapt software (included in the QIIME 2 suite). Trimmed sequences were error-filtered and truncated using the DADA2 software. Based on the quality score summary, R1 reads were truncated at 290 bp while R2 reads at 230 bp (Figures 2A,B). The resulting paired-end sequences were merged and searched for chimeras. The minimum abundance of potential ancestral sequences of a given merged sequence being tested as chimeric was increased to 8. An overview of DADA2 processing steps is given in Figure 2C. For comparison with existing Operational Taxonomic Unit (OTU) tables, open-reference OTU picking was applied against SILVA 123, using a threshold of 99% identity. Finally, taxonomy classification was performed with the RDP classifier. During this process, an assignment confidence cutoff of 80% was applied.

Meta-analysis of the QIIME 2 results was performed using R v4.2.0. BIOM files were processed using the {rbiom} package, while alpha diversity was calculated using the {vegan} package. Relative abundance analysis was performed with DESeq2 (Love et al., 2014). All plots were created using the {ggplot2} library (Wickham, 2016). The OTU table was annotated by keeping the genus labels from the RDP taxonomy results. Any OTUs (genus) or samples that have zero counts across the table were removed. In addition, samples with less than 10,000 reads were also removed from the rest of the analysis. All comparisons were performed at genus level taking into account the relative abundance of each genus in each sample.

Assessment of statistical significance was performed with the packages incorporated in the aforementioned bioinformatics tools.

## Results

### Comparison of cervicovaginal microbial composition between self-collected and physician-collected samples reveals significant overlap

In total, 38,328,108 raw reads (median 33,133 reads/sample) were obtained for the 937 sequenced PCR amplicons. After quality filtering, 30,353,253 non-chimeric reads (median 26,516 reads/sample) were further processed for taxonomic assignment and meta-data analysis (Figure 2C).

**Figure 3:**
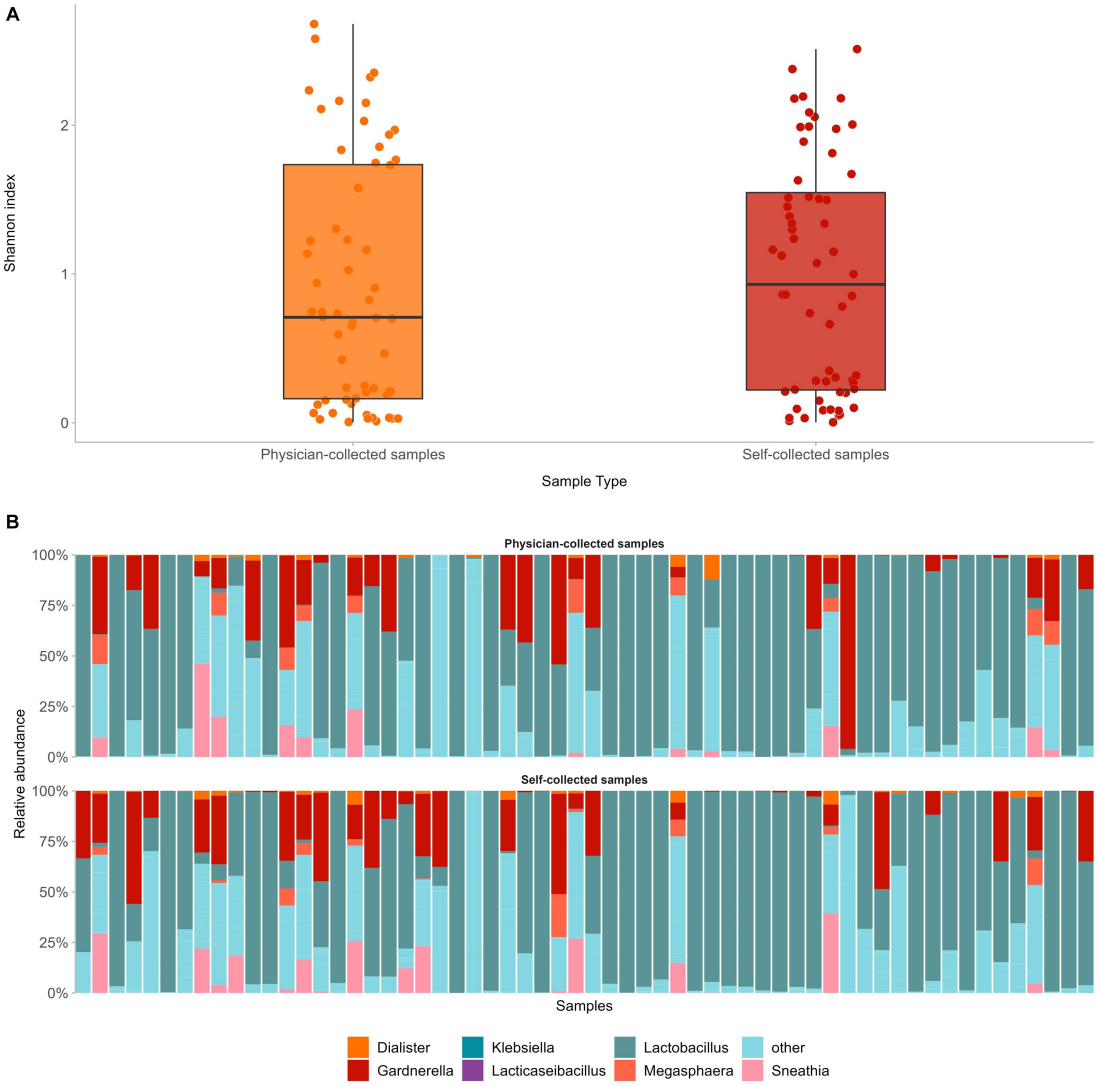
Comparison of paired self- versus physician-collected samples showed no significant differences in terms of vaginal microbial diversity **(A)**, as well as composition of the predominant bacterial genera in both sample types **(B)**.

First, we assessed how self-collected samples compared to the respective physician-collected ones in regard to microbial diversity and composition. To that end, we compared paired self- versus physician-collected samples from 60 women and compared alpha diversity and taxonomic assignment. Despite differences in the sampling methods, alpha microbial diversity did not differ significantly between the two sample types (median Shannon Index 0.9 and 0.7 for self-versus physician-collected samples, respectively, *p* = 0.4) (Figure 3A). In addition, in both sample types the same microbial populations dominated the cervicovaginal flora, with *Lactobacillus* being the predominant genus, followed by anaerobic, Community State Type IV (CST-IV) bacteria associated with bacterial vaginosis, such as *Gardnerella, Prevotella, Sneathia*, and *Megasphaera*. Altogether, the dominant genera in both samples accounted for an average 57.7% of the cervicovaginal microbiome, with each bacterial genus displaying similar relative frequencies in both the self- and the physician-collected samples (Figure 3B).

### Operational taxonomic unit assignment and comparison between different CIN groups and HPV types

Taxonomic assignment was performed with the RDP classifier, and vaginal microbial composition was compared in women infected with different hrHPV types, namely HPV16, HPV18, HPV other, as well as women who exhibited precancerous cervical lesions after abnormal colposcopic findings. For the latter comparison, groups were as defined above, i.e., CIN1, CIN2, CIN3, or, in the absence of any lesions, NDD (no detectable disease).

Overall, 10,788 bacterial operational taxonomic units (OTUs) were identified, of which 4,860 matched with 305 different and well-characterized genera. In all groups, we observed a *Lactobacillus*-dominated vaginal flora with a median relative abundance of 60%. Besides *Lactobacillus*, the majority of the vaginal microbiome in HPV-infected women consisted of CST-IV bacteria common in all groups irrespective of the HPV type responsible for the infection, such as *Gardnerella* (median frequency 11.5%), *Prevotella* (median frequency 3.8%), *Sneathia* and *Megasphaera* (median frequencies 1.8 and 0.6%, respectively), amongst others.

### Vaginal microbial diversity and richness is independent of the type of HPV or the presence of cervical lesions, however associates with age

**Figure 4:**
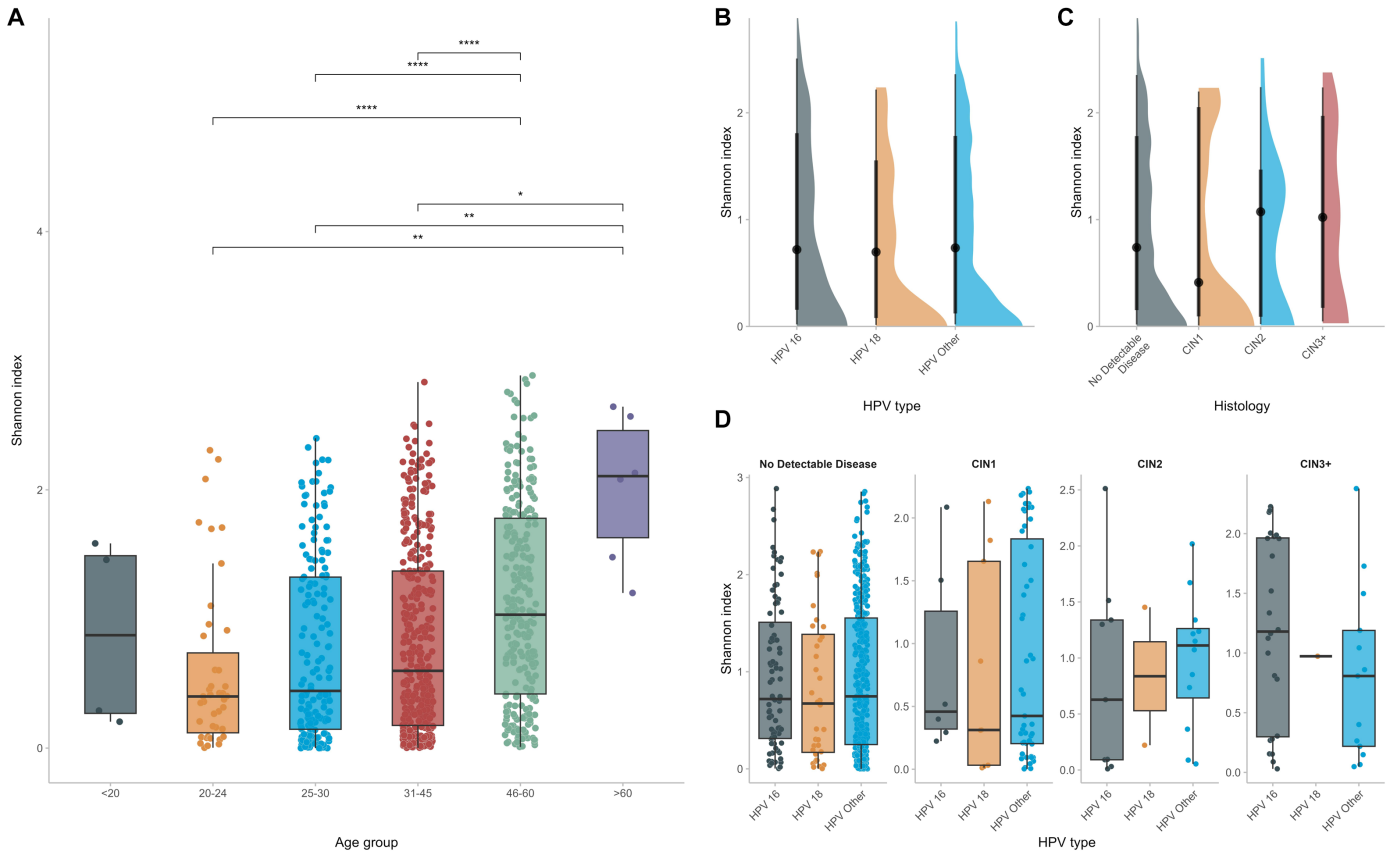
Alpha diversity was calculated and compared among different groups **(A)**. HPV infection does not affect significantly vaginal microbial diversity **(B–D)** whereas increased age seems to be the predominant factor for increased vaginal diversity.

Alpha diversity was calculated at the OTU level for the different groups with the Shannon Index. No significant differences were found in terms of microbial diversity for women infected with different HPV types. In fact, although CIN2 and CIN3 groups tended to be slightly more diverse than the CIN1 or NDD groups, such comparisons did not reach statistical significance. Indeed, the CIN2 and CIN3 groups displayed a higher Shannon index (Figure 4C), however comparison with the CIN1 or NDD groups did not reach statistical significance (*p* = 0.6), meaning that regardless the histopathology status, all women had a similar microbial diversity.

On the contrary, when considering the age of the study participants, the vaginal flora of women who were of 45 years of age or older was significantly more diverse than that of younger women (*p* < 0.0001), supporting that, even in the context of HPV, age at infection is the dominant determinant of vaginal diversity and richness (Figure 4A).

### HPV infection displays distinct vaginal microbial signatures compared among HPV types

**Figure 5:**
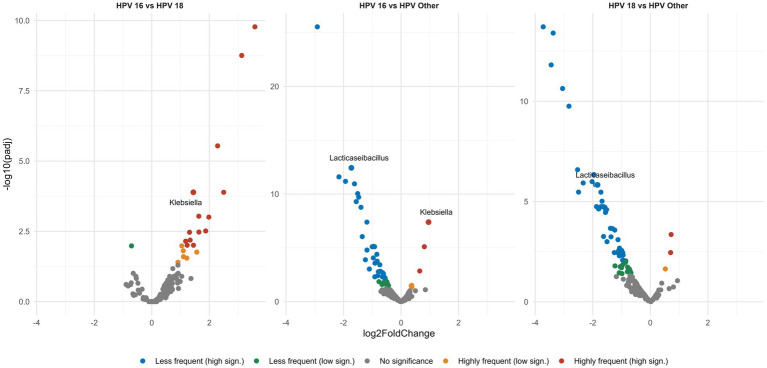
Volcano plots show pairwise comparisons of the relative abundance of the different genera. Each dot represents a specific genus and is plotted on a Cartesian coordinate system, where the *x* axis represents the fold change in the relative abundance between the compared groups and the *y* axis represents the statistical significance describing the low frequency (green, blue dots) or high frequency (orange, red dots) of each genus in a given group. All HPV-positive groups display significant *Lacticaseibacillus* depletion. Among the three HPV-positive groups the HPV16, 18 types show the greatest *Lacticaseibacillus* depletion of all.

Having identified the main bacterial genera that populate the vaginal microenvironment, next we explored if their relative abundance differed between the different groups. Differential analysis revealed pronounced differences in the abundance of certain bacterial genera between HPV types. In detail, all three HPV-positive groups exhibited increased frequencies of the CST-IV genera *Megasphaera* and *Sneathia*. Especially for *Lacticaseibacillus*, we observed a significant depletion in the case of types HPV16, HPV18 compared to cases infected with other high-risk HPV type(s) (*p* < 0.0001) (Figure 5). Similar comparisons were also performed among groups based on the grade of CIN in order to explore if the abundance of the aforementioned or other bacterial genera differed significantly in the presence of precancerous lesions: however, no significant differences were identified.

## Discussion

The increasingly evident role of the cervicovaginal microbiome in HPV infection persistence and cervical cancer pathogenesis alongside the diverse applications of high-throughput sequencing methodologies in metagenomics analyses, pose an exciting challenge for the use of NGS towards identifying microbial markers that will advance our understanding of HPV-associated cervical cancer ontogeny. Identifying specific bacterial species as microbial biomarkers of high-grade and lowgrade dysplasia could conceivably assist in triaging individuals with pronounced chances of lesion regression or progression (Kudela et al., 2021). In that regard, the assessment of microbial composition and associated risk factors constitutes the critical background for preventive, predictive, and personalized medicine (3P medicine) improving state-of-the-art medical care in patients with cervical cancer (Kudela et al., 2021).

In this context, we analyzed the vaginal and cervical microbial composition using high-throughput, amplicon-based metagenomics and investigated its relationship with HPV infection, using self- and physician-collected samples obtained from a large cohort of HPV positive women.

Comparison of paired self- versus physician-collected samples showed no significant differences in terms of either the vaginal microbial diversity or the composition of the predominant bacterial genera. The same microbial populations dominated the cervicovaginal flora, displaying similar relative frequencies in both self-collected and physician-collected specimens, with *Lactobacillus* being the predominant genus, followed by anaerobic bacteria, associated with bacterial vaginosis, such as *Gardnerella, Prevotella, Sneathia, Megasphaera* (Caselli et al., 2020; Wei et al., 2021; Lin et al., 2022). The aforementioned similarities between the two different sample types are noteworthy, especially in light of increasing evidence on the value of self-sampling as an equally informative approach for HPV testing as sampling by a healthcare professional. Our results reinforce this notion, showing for the first time that self-sampling can be implemented not only for HPV testing but also for other molecular-based applications, including microbiome analysis in the context of HPV infection.

Comparison of the vaginal microbiota between groups of women infected by different HPV types showed no significant differences in terms of microbial diversity, with *Lactobacillus* being the dominant genus in the vaginal flora of all groups. Similar to previous studies (Chen et al., 2020; Usyk et al., 2020; Wei et al., 2021), the majority of the vaginal microbiome in HPV-infected women consisted of CST-IV bacteria common in all groups irrespective of the HPV type responsible for the infection, revealing species assigned to *Gardnerella*, *Prevotella*, *Sneathia* and *Megasphaera* genera, amongst others. Previous studies have offered evidence that the presence of *Gardnerella* species in the vaginal microbiome is associated with progression to CIN2^+^ (Usyk et al., 2020; Xu et al., 2022). This might be attributed to the release of vaginal cytolysin and production of sialidase in the vaginal microenvironment, inducing tissue destruction, immune response evasion and bacterial invasion (Usyk et al., 2020; Xu et al., 2022). Similarly, other bacteria such as *Prevotella*, *Bacteroides* and *Mobiluncus* may inhibit the protective effect of the vaginal mucosal barrier by producing sialidase (Dong et al., 2022). The presence of these bacteria in our cohort further supports that HPV infections appear to go hand-in-hand with an overall dysbiotic vaginal microenvironment. However, it remains unclear whether it is the HPV infection itself that disrupts the balance of the vaginal microenvironment, or a pre-existing vaginal dysbiosis sets the ground for a persistent viral infection.

Although several reports mention an increased microbial diversity and richness of the vaginal flora in the context of HPV infections (Usyk et al., 2020; López-Filloy et al., 2022), we did not observe any significant differences in microbial diversity between groups of women infected by different hrHPV types, including women with precancerous cervical lesions. However, significant differences in the vaginal microbial diversity were observed depending on the age of HPV infected women. In fact, the vaginal flora of women who were of 45 years of age or older was significantly more diverse than that of younger women. This finding indicates that age is a major determinant of increased vaginal diversity, perhaps more significant than HPV infection status.

Finally, despite the overall similarities in the composition of the vaginal microbiome between the different examined groups, we found differences in the abundance of certain bacterial genera in the vaginal microenvironment among the HPV-infected women. In accordance with previous studies (Ravel et al., 2011; Zhou et al., 2019; Caselli et al., 2020), *Lacticaseibacillus* was significantly depleted in all HPV-positive women, while increased frequencies were also seen for the CST-IV genera *Megasphaera* and *Sneathia*. HPV infections can disturb the balance of vaginal and cervical microenvironment, leading to abnormal changes in microbiota, including *Lactobacillus* spp. depletion and predominance of *Lactobacillus iners* and anaerobic bacteria (such as *Gardnerella, Prevotella, Sneathia, Megasphaera*) (Lee et al., 2013; Bik et al., 2019; Borgogna et al., 2020; Caselli et al., 2020; Usyk et al., 2020; Wei et al., 2021; Lebeau et al., 2022), allowing the persistence of viral infection and disease progression (Li et al., 2012). Studies have demonstrated that abnormal changes in the composition of the vaginal microbiome, which lead to reduced mucus production and consequent clearance of pathogenic bacteria, can be associated with the late clearance of HPV infection (Li et al., 2012).

A major finding of the present study concerned the significant differences among different HPV types regarding the abundance of *Lacticaseibacillus*. In particular, *Lacticaseibacillus* was significantly depleted in the case of women infected by types HPV16, HPV18 compared to cases infected by other high-risk HPV type(s). This was particularly intriguing, especially in view of published evidence that a *Lacticaseibacillus* species, namely *Lacticaseibacillus casei* LH23 strains, inhibits the expression of the E6 and E7 viral proteins, which play a crucial role in viral replication during HPV infection (Tomaić, 2016). In this context, this novel finding could potentially be relevant in explaining the high risk for cervical cancer posed by the HPV16, 18 types. On the contrary, we found no significant differences in the abundance of certain bacteria (including the aforementioned genera) when comparing groups based on the presence or not of precancerous lesions. This indicates that although microbial profiling could provide insightful information regarding the clinical manifestations of certain hrHPV types, it appears as less helpful for triaging patients by risk of CIN/cancer development.

A limitation of our study concerns the lack of information regarding antibiotic usage by the study participants. This was due to the epidemiological nature of the GRECOSELF study which provided the samples. Although all participants stated that they were in good health and were not under any medication, we cannot *a priori* exclude the possibility of any alterations in the microbial composition due to potential antibiotic activity. Another limitation concerns the lack of a healthy control group consisting of women without an HPV infection, thus precluding the possibility to perform comparisons versus hr-HPV infected women. This was due to the retrospective nature of the study, whereby the analyzed samples were collected in the context of the GRECOSELF project (Agorastos et al., 2019), that investigated the feasibility of implementing secondary prevention of cervical cancer in Greece through HPV-DNA testing on self-collected cervicovaginal samples. In GRECOSELF, all samples that tested negative for HPV during GRECOSELF, were excluded from any downstream procedure and no material was stored for further use, eventually leading to the absence of a control group in the present study. Also worth mentioning is the fact that we were unable to assess the potential relationship between microbial composition and the cytological results in the women of our cohort. That was due the fact that in the context of the GRECOSELF study, upon confirmation of HPV infection, women were referred to their ob/gyn for a PAP smear. However, most participants did not follow up with their physician, thus leading to significant lack of information regarding the cytological results of our cohort, and hampering our ability to perform a robust statistical analysis regarding the correlation of the microbial composition and the cytological results.

In conclusion, we performed in-depth, high-throughput cervicovaginal microbiome profiling in one of the largest series of HPV infected women reported so far offering evidence for a distinct cervicovaginal microbial composition in HPV-infected women in relation to hrHPV types. Although our findings do not allow definitive conclusions to be drawn regarding their clinical relevance, still they provide the grounds for further exploration of links between differential cervicovaginal microenvironments and different types of HPV infection, their persistence and progression to cervical precancer and cancer.

## Data availability statement

The datasets presented in this study can be found in online repositories. The names of the repository/repositories and accession number(s) can be found at: https://www.ebi.ac.uk/ena, PRJEB59411.

## Ethics statement

The studies involving humans were approved by Ethics Committees of the Aristotle University of Thessaloniki and the Centre for Research and Technology Hellas. The studies were conducted in accordance with the local legislation and institutional requirements. The participants provided their written informed consent to participate in this study. Written informed consent was obtained from the individual(s) for the publication of any potentially identifiable images or data included in this article.

## Author contributions

ES: Formal analysis, Methodology, Writing – original draft. GG: Formal analysis, Methodology, Writing – original draft. NP: Formal analysis, Visualization, Writing – original draft. KP: Methodology, Writing – review & editing. KC: Investigation, Supervision, Writing – review & editing. FP: Formal analysis, Writing – review & editing. TA: Investigation, Supervision, Writing – review & editing. KS: Investigation, Supervision, Writing – review & editing.
